# CD96 Correlates With Immune Infiltration and Impacts Patient Prognosis: A Pan-Cancer Analysis

**DOI:** 10.3389/fonc.2021.634617

**Published:** 2021-02-19

**Authors:** Wenrui Ye, Cong Luo, Fangkun Liu, Zhixiong Liu, Fenghua Chen

**Affiliations:** ^1^ Department of Neurosurgery, Xiangya Hospital, Central South University (CSU), Changsha, China; ^2^ Clinical Medicine Eight-year Program, Xiangya Medical School of Central South University, Changsha, China; ^3^ Department of Urology, Xiangya Hospital, Central South University (CSU), Changsha, China; ^4^ National Clinical Research Center for Geriatric Disorders, Xiangya Hospital, Central South University (CSU), Changsha, China

**Keywords:** CD96, biomarker, cancer, bioinformatics, prognosis, immune infiltration

## Abstract

**Background:**

Immunotherapy has significantly improved patient outcomes, but encountered obstacles recently. CD96, a novel immune checkpoint expressed on T cells and natural killer (NK) cells, is essential for regulating immune functions. However, how CD96 correlating with immune infiltration and patient prognosis in pan-cancer remains unclear.

**Methods:**

HPA, TCGA, GEO, GTEx, Oncomine, TIMER2.0, PrognoScan, Linkedomics, Metascape, and GEPIA2 databases were used to analyze CD96 in cancers. Visualization of data was mostly achieved by R language, version 4.0.2.

**Results:**

In general, CD96 was differentially expressed between most cancer and adjacent normal tissues. CD96 significantly impacted the prognosis of diverse cancers. Especially, high CD96 expression was associated with poorer overall survival (OS) and disease-specific survival (DSS) in the TCGA lower grade glioma (LGG) cohort (OS, HR = 2.18, 95% CI = 1.79–2.66, *P* < 0.001). The opposite association was significantly observed in skin cutaneous melanoma (SKCM) cohort (OS, HR = 0.96, 95% CI = 0.94–0.98, *P* < 0.001). Notably, SKCM samples demonstrated the highest CD96 mutation frequency among all cancer types. Furthermore, in most cancers, CD96 expression level was significantly correlated with expression levels of recognized immune checkpoints and abundance of multiple immune infiltrates including CD8+ T cells, dendric cells (DCs), macrophages, monocytes, NK cells, neutrophils, regulatory T cells (Tregs), and follicular helper T cells (Tfh). CD96 was identified as a risk factor, protective factor, and irrelevant variable in LGG, SKCM and adrenocortical carcinoma (ACC), respectively. CD96 related genes were involved in negative regulation of leukocyte in LGG, however, involved in multiple positive immune processes in SKCM. Furthermore, CD96 was significantly associated with particular immune marker subsets. Importantly, it strongly correlated with markers of type 1 helper T cell (Th1) in SKCM, but not in LGG or ACC either.

**Conclusions:**

CD96 participates in diverse immune responses, governs immune cell infiltration, and impacts malignant properties of various cancer types, thus standing as a potential biomarker for determining patient prognosis and immune infiltration in multiple cancers, especially in glioma and melanoma.

## Introduction

In the past few decades, cancer has gradually become the top killer threatening human health (1). With the deepening understanding of the mechanism underlying cancer initiation and development, we have opened up more options to fight it (2). Co-inhibitory or immune checkpoint receptors, such as cytotoxic T lymphocyte associated protein 4 (CTLA-4) and programmed death-1 (PD-1), are expressed on immune cells to limit the immune responses, and prevent immune-driven pathology. Indeed, immunotherapies blocking these receptors have shown tremendous success in the treatment of several cancers. However, despite the great success of immune checkpoint blockade (ICB), a considerable number of patients still do not respond to currently available immunotherapies (3). Therefore, the dilemma has attracted attention to exploring new immune checkpoints that can be safely targeted with high anti-tumor efficacy in malignancies, with the hope that targeting more co-inhibitory receptors will lead to higher response rates and better therapeutic outcomes.

Previous review has elucidated the mechanism of CD226/TIGIT/CD96 pathway, addressing the important role of these membrane-sided signal receptors in multiple cancer types (4). Especially, TIGIT (T-cell immunoglobulin and ITIM domain) can suppress immune responses by counterbalancing costimulatory receptor CD226-mediated immune activation *via* hampering CD155-CD226 interaction, since TIGIT harbors higher affinity with CD155 than CD226 (5). TIGIT also exerts certain effects on the tolerogenic DC phenotype and Treg stability (6, 7). These properties of TIGIT enhance the immunosuppressive functions, partially contributing to immune escape of malignant tumors. Emerging evidence confirms the combination of PD-L1 and TIGIT blockades (atezolizumab/tiragolumab) as a promising approach for targeting tumors resistant to a single ICB.

Involved in this pathway, CD96 is regarded as a novel immune checkpoint and suspected harboring potential in the context of immunotherapy. However, data to date revealed seemingly controversial results. Study has suggested that CD96 could synergize with TIGIT to inhibit the antitumor response in tumor-bearing mouse models with lung metastasis, as the antitumor effect of CD96 blockade is higher in *Tigit* ^−/−^ mice (8). Anti-CD96 monoclonal antibody (mAb) also demonstrates higher efficacy in combination with either anti-CTLA-4 or anti-PD-1 mAbs, depending on the activation of CD226 signaling in NK cells (9, 10). However, CD96 paradoxically acts as a costimulatory receptor and activates CD8+ T cells (11), making it still uncertain whether CD96 functions as an immunosuppressive receptor. A more comprehensive analysis of CD96 profile in human cancer is warranted understand immune-cell-intrinsic effect of CD96 in tumor immunity. We herein conducted a pan-cancer analysis to illustrate the CD96 profiles including expression, mutation status, correlation with signatures of interest, as well as contribution to patient’s survival. In this study, all data was elicited from well-known open databases, and all these analyses were conducted based on webtools and R language.

## Results

### CD96 Expression Profiles in Human Normal Tissues

**Figure 1:**
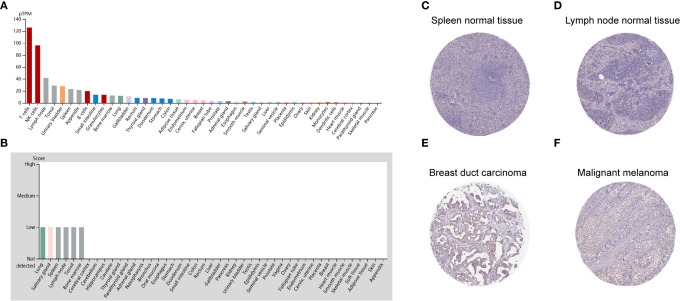
CD96 expression profiles in human normal and cancer tissues. **(A)** CD96 expression profiles in normal human tissues. **(B)** The protein expression profiles of CD96 in human normal tissues. **(C–F)** Representative IHC images of CD96 expression in normal lymph node tissues, normal spleen tissues, breast duct carcinoma tissues, and malignant melanoma tissues.

To detect the CD96 mRNA and protein expression profiles in human tissues, we evaluated the expression of CD96 in various tumor and normal tissues using the Human Protein Atlas (HPA) database. As shown in **Figure 1A**, the CD96 mRNA expression was group enriched in blood and lymphoid tissues. We then examined the CD96 protein expression and found it widely expressed, but at low levels in various normal tissues (**Figure 1B**). Immunohistochemistry (IHC) showed CD96 protein was mainly distributed in cytoplasm and membrane, and was low expressed in non-germinal center cells in normal lymph node tissues and white pulp cells in normal spleen tissues (**Figures 1C, D**). Meanwhile, CD96 was also low expressed in several cancers, such as breast cancer and skin melanoma (**Figures 1E, F**). The detailed information of IHC results were summarized in **Table 1**.

**Table 1:** Clinical information and relative scores of immunohistochemistry results (IHC, immunohistochemistry).

Protein	Tissue	Histological type	Age	Gender	Location	Quantity	Intensity	Relative IHC score
CD96	Lymph node	Normal tissue	55	Male	Cytoplasmic/membranous	<25%	Weak	1
CD96	Spleen	Normal tissue	57	Male	Cytoplasmic/membranous	<25%	Weak	1
CD96	Breast cancer	Duct carcinoma	38	Female	Cytoplasmic/membranous	50–75%	Moderate	6
CD96	Melanoma	Malignant melanoma	77	Male	Cytoplasmic/membranous	50–75%	Moderate	6

### CD96 Expression Profiles in Human Cancers

CD96 mRNA distribution showed low cancer specificity. Furthermore, CD96 protein expression was extremely low in all cancer tissues, with moderate CD96 cytoplasmic positive expression observed only in a subset of lymphoid cells within the tumor stroma.

**Figure 2:**
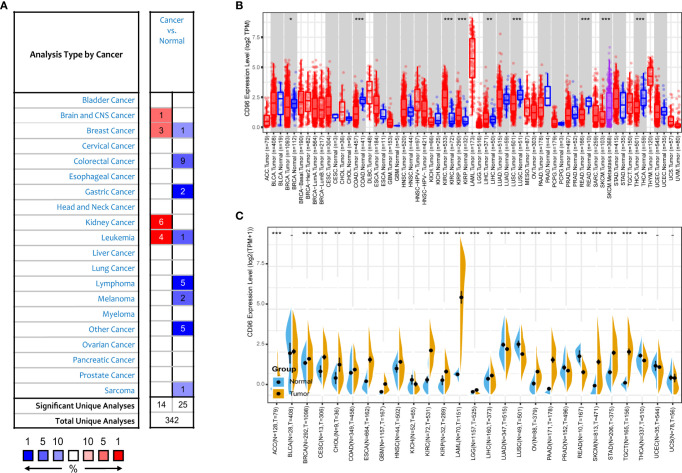
CD96 expression levels in different types of human cancers. **(A)** Increased or decreased CD96 in datasets of different cancers compared with normal tissues in the Oncomine database. **(B)** CD96 expression levels in different tumor types from TCGA database were analyzed by TIMER2.0 (*P < 0.05, **P < 0.01, ***P < 0.001). **(C)** Comparisons of CD96 expression levels between tumor tissues from TCGA database and normal tissues from GTEx database (*P < 0.05, **P < 0.01, ***P < 0.001).

We next compared expression differences of CD96 mRNA in cancers and normal tissues using Oncomine database, and found it expressed at relatively higher levels in brain cancer, breast cancer, renal cancer, and leukemia than in normal tissues (**Figure 2A**). But in certain studies, CD96 was less expressed in breast cancer, colorectal cancer, gastric cancer, and leukemia, lymphoma, melanoma, and sarcoma. To further evaluate the differential expression of CD96, we compared the its expression levels in the TCGA dataset using TIMER2.0. As shown in **Figure 2B**, CD96 expression was significantly elevated in various cancer types, including esophageal carcinoma (ESCA), head and neck squamous cell carcinoma (HNSC), kidney renal clear cell carcinoma (KIRC), kidney renal papillary cell carcinoma (KIRP), and stomach adenocarcinoma (STAD). However, the expression of CD96 in breast invasive carcinoma (BRCA), colon adenocarcinoma (COAD), lung squamous cell carcinoma (LUSC), rectum adenocarcinoma (READ), SKCM, and thyroid carcinoma (THCA) was significantly decreased.

The number size of normal tissue in the TCGA database is too small to be statistically convincing [e.g., TCGA glioblastoma multiforme (GBM) cohort has only five normal controls], so we matched the GTEx normal tissues with the TCGA cancer tissues to reflect the CD96 expression landscape in a more convincing manner, and found that CD96 was differentially expressed in most cancers (**Figure 2C**). Specifically, the CD96 expression level was significantly increased in ACC, BRCA, endocervical adenocarcinoma (CESC), cholangiocarcinoma (CHOL), COAD, ESCA, GBM, HNSC, KIRC, KIRP, acute myeloid leukemia (LAML), LGG, liver hepatocellular carcinoma (LIHC), ovarian serous cystadenocarcinoma (OV), pancreatic adenocarcinoma (PAAD), SKCM, STAD, and testicular germ cell tumors (TGCT). On the contrary, CD96 was low expressed in lung adenocarcinoma (LUAD), LUSC, READ, and THCA compared to GTEx normal controls.

### The Association Between CD96 Expression and Cancer Patient’s Prognosis

**Figure 3:**
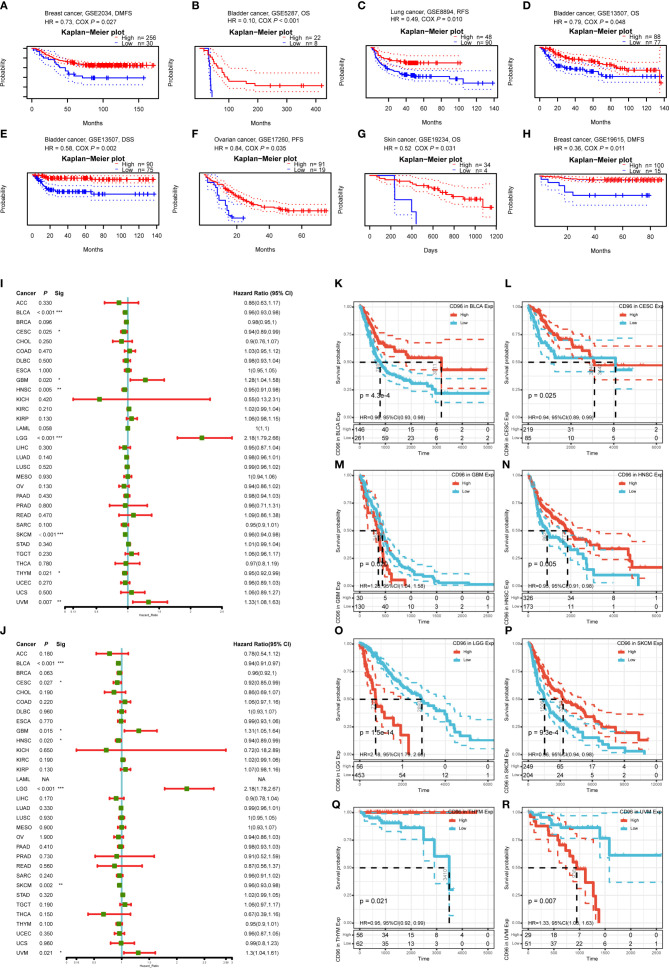
Survival analysis comparing the high and low expression of CD96 in different types of cancer in the GEO dataset and TCGA dataset. **(A–H)** Survival curves in eight cohorts (GSE5287, GSE13507, GSE19615, GSE2034, GSE17537, GSE8894, GSE17260, and GSE19234) with significance. **(I, J)** Relation between CD96 expression and patient prognosis (OS and DSS) of different cancers in TCGA database (*P < 0.05, **P < 0.01, ***P < 0.001). **(K–R)** Survival curves of OS with significance in eight cancer types (BLCA, CESC, GBM, HNSC, LGG, SKCM, THYM, and UVM) in TCGA.

To understand how CD96 impacting cancer patient prognosis, we used the PrognoScan database to analyze the relationship between CD96 and the survival outcomes of cancer patients. Results based on eight cohorts [GSE5287 (12), GSE13507 (13, 14), GSE19615 (15), GSE2034 (16), GSE17537 (17–20), GSE8894 (21), GSE17260 (22), GSE19234 (23)] suggested that high expression of CD96 was significantly associated with better prognosis (COX *P* < 0.05; **Figures 3A–H**). Detailed information of these cohorts can be found in **Supplementary Table 1**.

To further examine the prognostic potential of CD96, we used TCGA RNA-seq and clinical data downloading from UCSC Xena to analyze the prognosis of 33 TCGA cancer types. As shown in **Figure 3I**, elevated CD96 expression was significantly related to a poorer OS in GBM (HR = 1.28, 95% CI = 1.04–1.58, *P* = 0.020), LGG (HR = 2.18, 95% CI = 1.79–2.66, *P* = 1.5e-14), and uveal melanoma (UVM; HR = 1.33, 95% CI = 1.08–1.63, *P* = 0.007). On the contrary, increased CD96 expression was associated with the better prognosis in BLCA (HR = 0.96, 95% CI = 0.93–0.98, *P* = 4.3e-4), CESC (HR = 0.94, 95% CI = 0.89–0.99, *P* = 0.025), HNSC (HR = 0.95, 95% CI = 0.91–0.98, *P* = 0.005), SKCM (HR = 0.96, 95% CI = 0.94–0.98, *P* = 9.3e-4), and thymoma (THYM) (HR = 0.95, 95% CI = 0.92–0.99, *P* = 0.021). The survival curves with significance (*P* < 0.05) were displayed as **Figures 3K–R**. Furthermore, to avoid the bias resulting from non-cancer events, DSS was analyzed and shown as **Figure 3J**. The result, much like that of the OS analysis, indicated higher CD96 expression to be significantly related to a poorer DSS in GBM (HR = 1.31, 95% CI = 1.05–1.64, *P* = 0.015), LGG (HR = 2.18, 95% CI = 1.78–2.67, *P* = 6.7e-14), and UVM (HR = 1.30, 95% CI = 1.04–1.61, *P* = 0.021). In contrast, low CD96 expression was related to a poorer DSS in BLCA (HR = 0.94, 95% CI = 0.91–0.97, *P* = 1.6e-4), CESC (HR = 0.92, 95% CI = 0.85–0.99, *P* = 0.027), HNSC (HR = 0.94, 95% CI = 0.84–0.99, *P* = 0.020), and SKCM (HR = 0.96, 95% CI = 0.93–0.98, *P* = 0.002). These results clearly demonstrated that the CD96 expression was significantly associated with patient prognosis in multiple cancer types.

### The Landscape of CD96 Mutation Profile in Different Tissues

**Figure 4:**
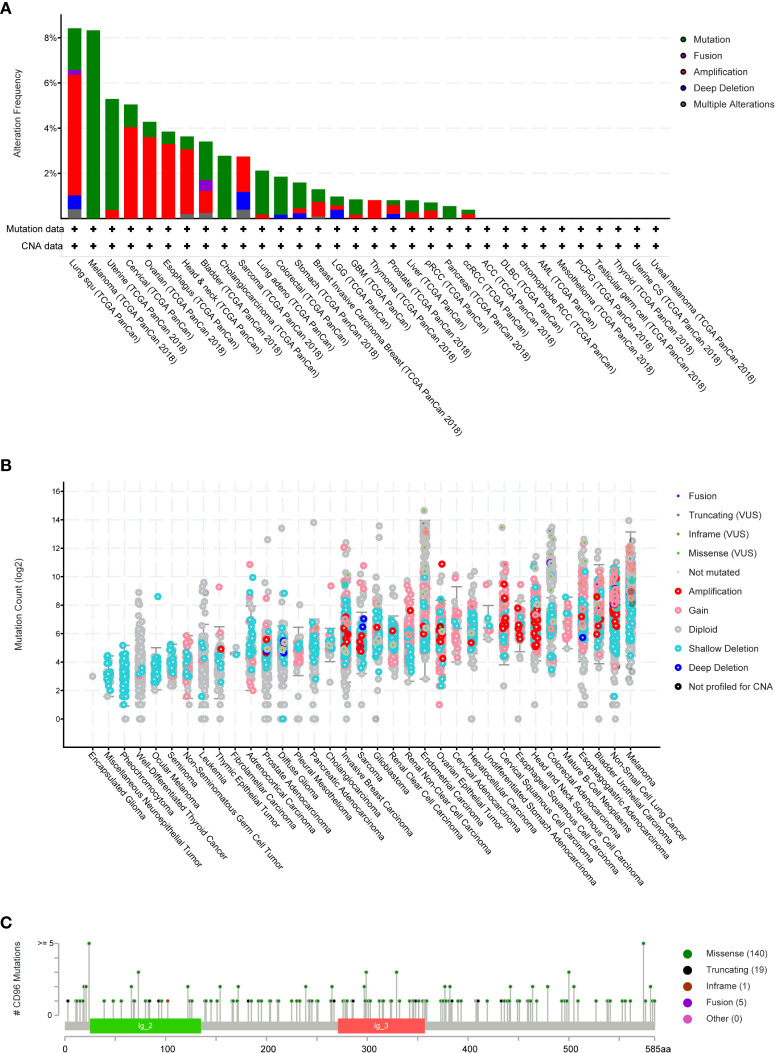
CD96 mutation landscape. **(A)** CD96 mutation frequency in multiple TCGA pan-cancer studies according to the cBioPortal database. **(B)** The general mutation count of CD96 in various TCGA cancer types by the cBioPortal database. **(C)** Mutation diagram of CD96 in different cancer types across protein domains.

We then employed cBioPortal to inspect the mutation frequency of CD96 in the TCGA database (10967 samples in 32 studies), and we found that LUSC and SKCM shared relatively high mutation level with the CD96 alteration frequency exceeding 8% (**Figures 4A, B**). A total of 165 mutation sites (including 140 missense, 19 truncating, 1 inframe, and 5 fusion mutations) were detected locating between amino acids 0 and 585 (**Figure 4C**). Among them, E24K and E574K were the two most frequent mutation sites.

COSMIC provided detailed information on the CD96 mutation types, including substitution missense, non-sense, and synonymous mutations in different cancers. The results were depicted in pie charts (**Supplementary Figure 1**). Non-sense substitutions were found in cervix cancer (25%), large intestine cancer (2.47%), lung cancer (4.90%), and skin cancer (0.58%), while missense substitutions were observed in biliary tract cancer (5.56%), breast cancer (8.94%), cervix cancer (25%), central nervous system (CNS) cancer (33.33%), endometrial cancer (41.67%), hematopoietic and lymphoid cancer (7.41%), kidney cancer (25%), large intestine cancer (35.80%), liver cancer (8.70%), lung cancer (34.31%), esophageal cancer (25.53%), ovary cancer (12.50%), pancreas cancer (6.25%), prostate cancer (3.28%), skin cancer (44.77%), stomach cancer (16.22%), thyroid cancer (100%), upper aerodigestive tract cancer (46.15%), and urinary tract cancer (53.33%). Additionally, synonymous substitution mutations were detected in breast cancer (1.63%), CNS cancer (6.06%), endometrial cancer (22.22%), kidney cancer (4.17%), large intestine cancer (12.35%), liver cancer (4.35%), lung cancer (8.82%), skin cancer (12.79%), stomach cancer (10.81%), and urinary tract cancer (20%). Besides, C > T and G > A types were predominantly observed in the CD96 coding strand mutations. Other types of base mutations occurred sporadically in different cancers.

### Genome-Wide Association of CD96 mRNA in Cancer

**Figure 5:**
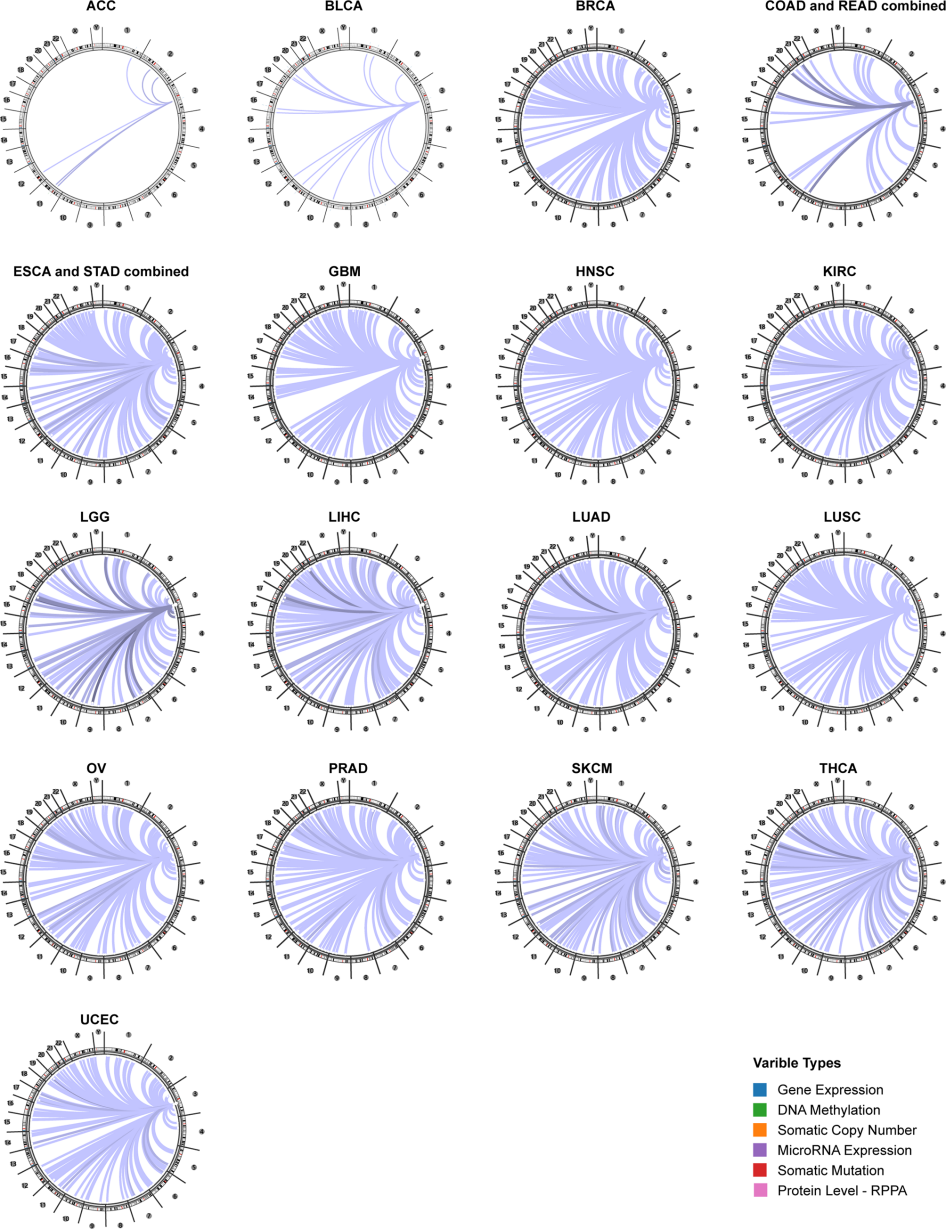
The genome-wide correlation between CD96 and other signatures from the TCGA database (Cancer Regulome program).

Using the Regulome Explorer web tool, we further explored the relevant genomic correlations between certain signatures and CD96. Based on the associations among gene, deoxyribonucleic acid (DNA) methylation, somatic copy number, somatic mutation and protein level, circus plots were displayed to illustrate these interrelations in human cancers. According to the data from TCGA, associations could be detected between CD96 and other signatures in ACC, BLCA, BRCA, COAD, READ, ESCA, STAD, GBM, HNSC, KIRC, LGG, LIHC, LUAD, LUSC, OV, prostate adenocarcinoma (PRAD), READ, SKCM, STAD, THCA, and uterine corpus endometrial carcinoma (UCEC) within the context of genomic coordinates (**Figure 5**). Detailed data can be found in **Supplementary Table 2**.

### Relationship Between Immunotherapy, Immune Checkpoints, and CD96

**Figure 6:**
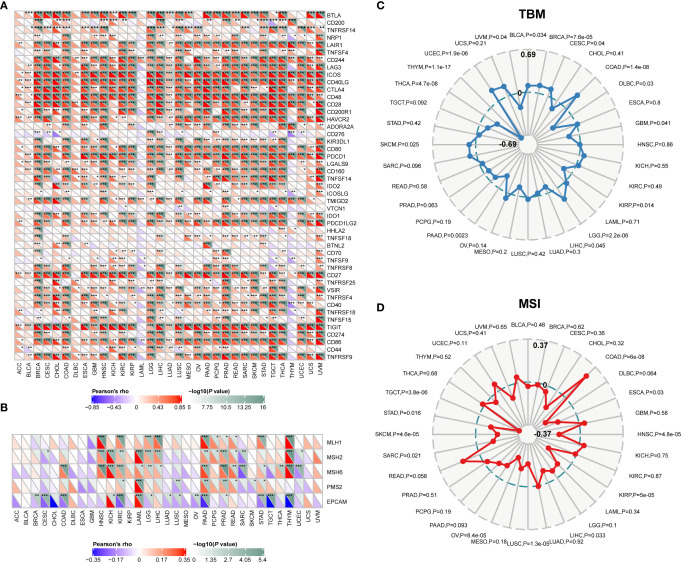
Correlations between CD96 and immune checkpoints, as well as other variables of interest. **(A)** The correlations between CD96 and confirmed immune checkpoints in multiple cancers (*P < 0.05, **P < 0.01, ***P < 0.001). **(B)** The correlations between CD96 and essential genes involved in MMR in multiple cancers (*P < 0.05, **P < 0.01, ***P < 0.001). **(C, D)** The correlations of CD96 expression and TMB, MSI in cancers.

The pan-cancer correlations between CD96 and immune checkpoints were displayed as **Figure 6A**. In most cancers, except ACC, BLCA, diffuse large B-cell lymphoma (DLBC), LAML, LUAD, and THYM, robust and significant relationships existed between CD96 expression and expression levels of recognized immune checkpoints including B- and T-lymphocyte attenuator (BTLA), leukocyte-associated immunoglobulin-like receptor 1 (LAIR1), CD244, lymphocyte activation gene 3 (LAG3), inducible T cell costimulator (ICOS), CD40 ligand (CD40LG), CTLA4, CD48, CD28, CD200 receptor 1 (CD200R1), CD80, PDCD1, transmembrane and immunoglobulin domain containing 2 (TMIGD2), programmed cell death 1 ligand 2 (PDCD1LG2), CD27, TIGIT, CD86 and tumor necrosis factor receptor superfamily 9 (TNFRSF9). This suggested a potential synergy of CD96 with known immune checkpoints. However, based on several published works summarized in TISIDB, there was no significant difference in expression of CD96 between responders and non-responders to immunotherapy (**Supplementary Table 3**).

Mismatch repair pathway plays a critical role in identifying and repairing mismatched bases during DNA replication and genetic recombination (24). DNA mismatch repair deficiency and subsequent microsatellite instability (MSI), a hypermutator phenotype secondary to frequent polymorphism in short repetitive DNA sequences and single nucleotide substitution (25), lead to the accumulation of mutation loads in cancer-related genes and the aggravation of tumor mutation burden (TMB) (26). They are responsible for tumor initiation and regarded as independent predictors of ICB efficacy (25, 27, 28). Here we examined the correlation between CD96 expression and several essential MMR signatures. CD96 expression was positively correlated with MutL homolog 1 (MLH1), MutS homolog 2 (MSH2), and MutS homolog 6 (MSH6) in HNSC, KIRC, LGG, LIHC, PAAD, PRAD, and THYM. In contrast, it was negatively correlated with epithelial cell adhesion molecule (EpCAM) in BRCA, CESC, COAD, KIRC, LAML, PAAD, STAD, TGCT, and THYM (**Figure 6B**). In addition, CD96 expression was positively correlated with TMB in COAD, LGG, and UCEC (*P* < 0.001), while negatively correlated with TMB in THCA and THYM cohorts (*P* < 0.001; **Figure 6C**). In general, MSI-High tumors were showed to express higher level of CD96 than genetically stable ones (*P* < 0.001), while the opposite trend existed in HNSC, KIRP, LUSC, OV, SKCM, and TGCT cohorts (*P* < 0.001, **Figure 6D**). Despite the significances of these correlations, the correlation coefficients between CD96 and TMB, as well as MSI, were below 0.6 in almost all cancers, suggesting that CD96 was rather unlikely to affect tumorigenesis by participating in the process of genetic alterations, and was not sufficient to independently predict the patient’s response to ICBs either.

### High CD96 Expression Correlates With Immune Infiltration in Cancer

**Figure 7:**
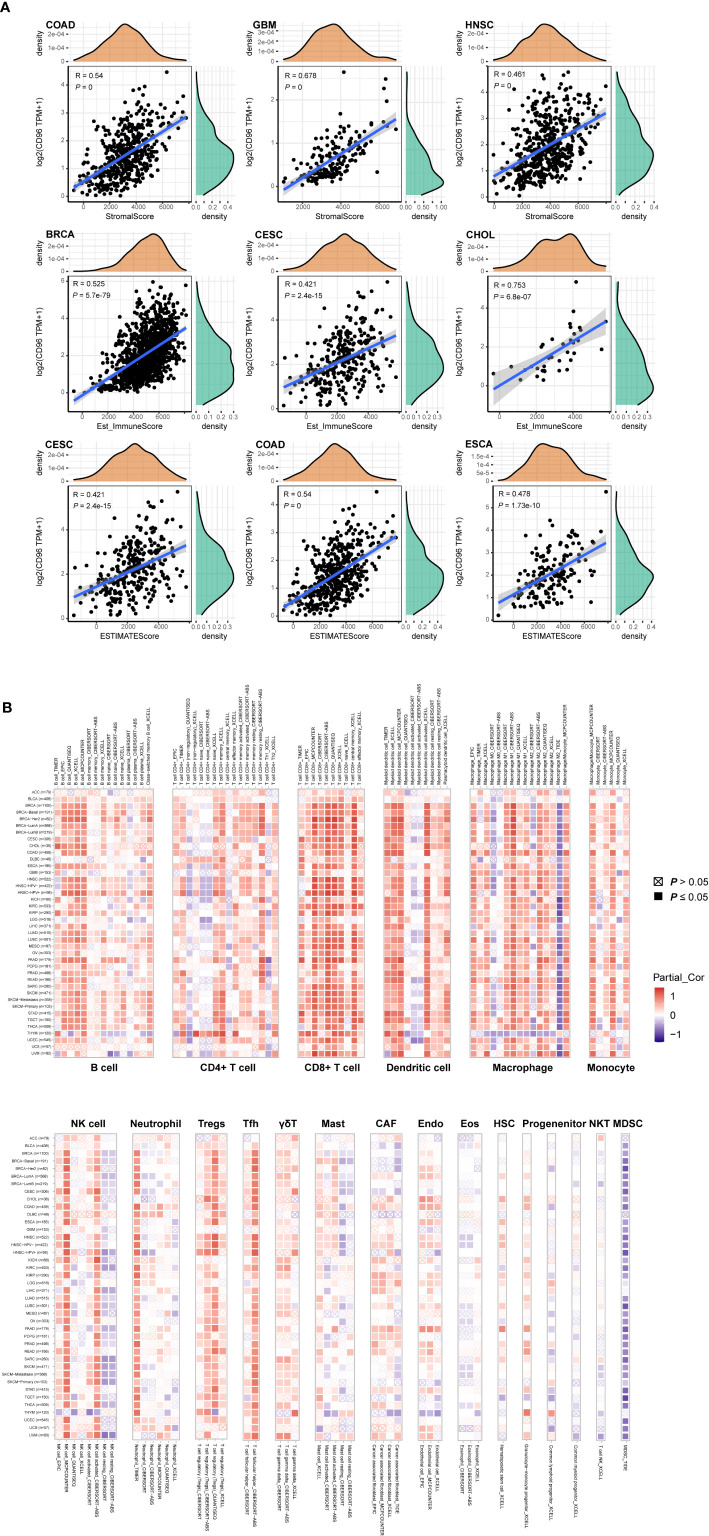
Associations of CD96 expression to tumor purity and immune infiltration. **(A)** Top three scatter plots of correlation between CD96 and stromal score, immune score, ESTIMATE score in various cancers. **(B)** The correlations of CD96 expression and immune infiltration in cancers.

To explore whether CD96 is involved in the process of immune infiltration in pan-cancer, we first evaluated the association between CD96 expression and tumor purity. As **Figure 7A** indicated, CD96 was most significantly associated with stromal scores in COAD, GBM, and HNSC. Meanwhile, the abundances of immune components in BRCA, CESC, and CHOL were significantly correlated with the expression level of CD96. Finally, the relationships between CD96 expression and tumor purity were significant in CESC, COAD, and ESCA. It suggested that CD96 was highly involved in the process of immune infiltration and formation of pluralistic components in the above tumors.

Besides, we employed TIMER2.0 to exhibit the landscape of CD96 correlating with various immune infiltrates in human cancers (**Figure 7B**). Overall, it was positively correlated with immune infiltrating levels of multiple infiltrates including CD8+ T cells, DCs, macrophages, monocytes, NK cells, neutrophils, Tregs, and Tfh. However, the negative correlation was discovered between CD96 expression and myeloid-derived suppressor cells (MDSC) abundance. The profile indicated that CD96, to some extent, participated in the immune infiltration process and played a vital role in the immuno-oncological interactions. It is worth noting that in tumors such as ACC, BLCA, DLBC, GBM, LGG, THYM, and uterine carcinosarcoma (UCS), the trend of this correlation was subtly different, which may be caused by the various immune infiltration ratios in different cancers. For instance, in brain tumors (GBM and LGG), CD96 was only positively correlated with infiltration levels of CD8+ T cells, dendritic cells, macrophages, and neutrophils, partially due to the distinct tumor microenvironments in central nervous system.

### CD96 Impacts Patient Prognosis *via* Intervening in Tumor Immunity

**Figure 8:**
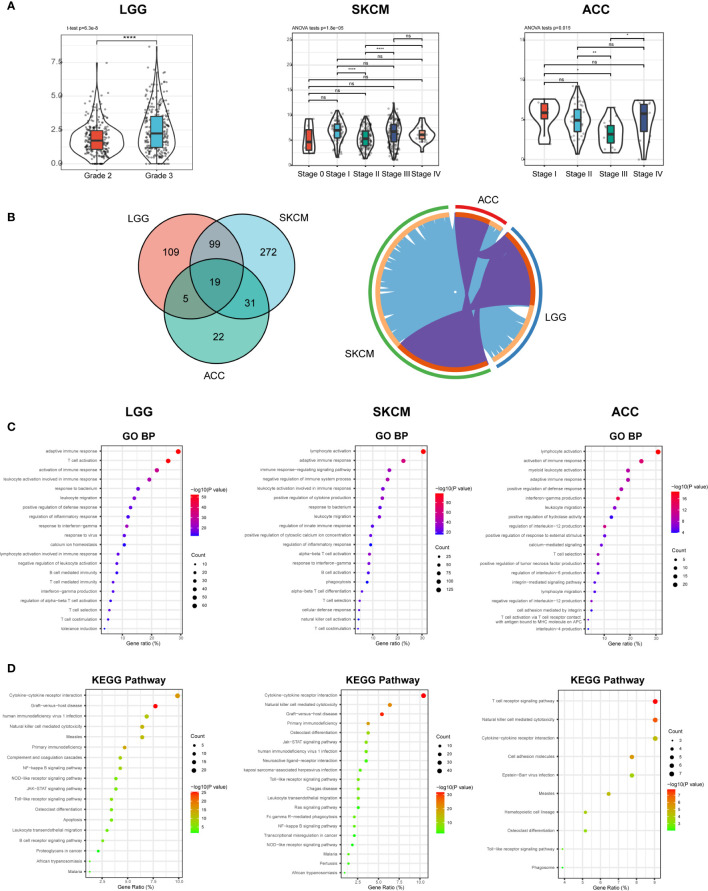
CD96 expression and function profiles in three representative cancers. **(A)** CD96 expression levels in different grades or stages in LGG, SKCM, and ACC, respectively (*P < 0.05, **P < 0.01, ***P < 0.001). **(B)** 232, 421, and 77 related genes were identified in LGG, SKCM, and ACC cohorts. Circos plot showed overlaps in genes (purple curves) and enriched ontology terms (blue curves) between three lists based on their functions or shared pathways. **(C, D)** GO and KEGG analysis of CD96-related signatures in LGG, SKCM, and ACC.

According to the expression, survival, and mutation analysis, we observed significant but contradictory roles of CD96 in different cancers. Considering that CD96 was a significant risk factor for LGG and a distinct protective factor for SKCM, we identified LGG and SKCM as representative cancer types for subsequent analysis, with ACC serving as a control group since CD96 seemingly had no impact on ACC prognosis. The subtype analysis revealed contrary expression distributions of CD96 in glioma and melanoma. Specifically, in LGG, CD96 expression in grade 3 was significantly higher than that in grade 2 (*P* < 0.001). In contrast, higher CD96 expression level was observed in lower stage of SKCM (*P* < 0.001). In addition, the differences in expression between different ACC stages were not so significant (**Figure 8A**).

To investigate the biological characteristics associated with CD96 in these representative cancers, we ranked the related genes using the Linkedomics website. In general, 232, 421, and 77 CD96 related genes were identified in LGG, SKCM, and ACC cohorts respectively (|R| > 0.6, *P* < 0.001; **Supplementary Table 4**). We performed functional analysis of these genes using the Metascape website and found that the three gene lists shared multiple overlapping genes and enriched terms (**Figure 8B**). Specifically, CD96 related genes were mostly associated with various immune responses (**Figure 8C**). Intriguingly, CD96 related genes in LGG were involved in negative regulation of leukocyte. However, genes in SKCM were intensively involved in positive immune processes including lymphocyte activation, NK cell activation, and positive regulation of cytokine production. This intriguing difference suggested that CD96 mediated immunosuppressive effects in glioma patients, but participated in completely opposite immune processes in melanoma patients, highly indicating that CD96 impacted patient prognosis *via* an immune-related manner. Incidentally, in ACC, CD96 related genes were enriched in terms that were not tightly linked to the typical or specific tumor immune response. Meanwhile, KEGG pathway enrichment analysis showed similar results, these genes were closely involved in a variety of immune processes, including cytokine-cytokine receptor interaction, NK cell mediated cytotoxicity, and nuclear factor kappa-B (NF-κB) signaling pathway, et al. (**Figure 8D**).

To clarify the specific cell types modulated by CD96 in tumor microenvironment (TME), we explored the correlations between CD96 expression and immune infiltrating levels in LGG and SKCM based on sets of immunological markers using the TIMER2.0 database, with ACC serving as a control cohort. We adjusted these results based on tumor purity, revealing strong and significant correlations between CD96 and CD8+ T cell markers (CD8A), general T cell markers (CD3D, CD3E, CD2), DC markers (HLA-DPB1, HLA-DRA, HLA-DPA1) in LGG. While in SKCM cohort, CD96 expression was significantly correlated with CD8+ T cell markers (CD8A, CD8B), general T cell markers (CD3D, CD3E, CD2), monocyte markers (CD86, CD115), DC markers (HLA-DPB1, HLA-DRA, HLA-DPA1), Th1 markers (TBX21, STAT1, STAT4, IFN-γ), Treg markers (FOXP3, CCR8), and exhausted T cell makers (PD-1, LAG3, TIM-3, GZMB) (Pearson’s rho > 0.6, *P* < 0.001). Besides, no significant correlation was detected between CD96 expression and any immune marker set in ACC cohort (**Table 2**). Further re-examination using the GEPIA2 database revealed consistent results (**Table 3**).

**Table 2:** Correlation analysis between CD96 and relate genes and markers of immune cells in TIMER2.0.

Description	Gene markers	LGG (n = 516)				SKCM (n = 471)				ACC (n = 79)			
		None		Purity		None		Purity		None		Purity	
		rho	*P*	rho	*P*	rho	*P*	rho	*P*	rho	*P*	rho	*P*
**CD8+ T cell**	CD8A	0.649	***	0.623	***	0.873	***	0.804	***	0.541	***	0.404	***
	CD8B	0.491	***	0.447	***	0.871	***	0.798	***	0.466	***	0.310	0.008
**T cell**	CD3D	0.794	***	0.779	***	0.883	***	0.813	***	0.482	***	0.321	0.006
**(general)**	CD3E	0.837	***	0.824	***	0.881	***	0.810	***	0.547	***	0.421	***
	CD2	0.839	***	0.827	***	0.903	***	0.845	***	0.525	***	0.371	0.001
**B cell**	CD19	0.386	***	0.339	***	0.695	***	0.582	***	0.065	0.567	0.014	0.908
	CD79A	0.284	***	0.281	***	0.715	***	0.589	***	0.287	0.010	0.128	0.282
**Monocyte**	CD86	0.448	***	0.408	***	0.827	***	0.732	***	0.446	***	0.237	0.044
	CD115 (CSF1R)	0.268	***	0.198	***	0.737	***	0.614	***	0.551	***	0.338	0.003
**TAM**	CCL2	0.460	***	0.426	***	0.580	***	0.409	***	0.410	***	0.216	0.066
	CD68	0.468	***	0.440	***	0.444	***	0.255	***	0.475	***	0.299	0.010
	IL10	0.444	***	0.422	***	0.638	***	0.503	***	0.342	0.002	0.085	0.472
**M1 Macrophage**	INOS (NOS2)	−0.046	0.305	−0.059	0.218	0.014	0.765	0.007	0.879	0.138	0.225	0.064	0.588
	IRF5	0.399	***	0.354	***	0.630	***	0.453	***	0.567	***	0.459	***
	COX2 (PTGS2)	0.308	***	0.271	***	0.102	0.028	0.032	0.501	0.239	0.034	0.099	0.403
**M2 Macrophage**	CD163	0.435	***	0.424	***	0.646	***	0.523	***	0.549	***	0.400	0.001
	VSIG4	0.285	***	0.240	***	0.601	***	0.475	***	0.522	***	0.321	0.006
	MS4A4A	0.424	***	0.420	***	0.704	***	0.583	***	0.502	***	0.269	0.021
**Neutrophils**	CD66b	0.032	0.493	0.032	0.510	−0.023	0.614	0.008	0.861	−0.113	0.320	−0.133	0.261
	CD11b (ITGAM)	0.388	***	0.334	***	0.648	***	0.534	***	0.551	***	0.349	0.002
	CCR7	0.599	***	0.585	***	0.793	***	0.670	***	0.628	***	0.544	***
**NK cell**	KIR2DL1	0.153	***	0.173	***	0.408	***	0.273	***	0.318	0.004	0.193	0.102
	KIR2DL3	0.301	***	0.307	***	0.577	***	0.419	***	0.237	0.036	0.134	0.260
	KIR2DL4	0.369	***	0.369	***	0.700	***	0.570	***	0.159	0.162	0.054	0.647
	KIR3DL1	0.198	***	0.192	***	0.553	***	0.408	***	0.298	0.008	0.219	0.063
	KIR3DL2	0.178	***	0.190	***	0.661	***	0.519	***	0.159	0.162	0.045	0.703
	KIR3DL3	0.019	0.676	0.033	0.498	0.212	***	0.165	***	0.300	0.007	0.155	0.191
	KIR2DS4	0.294	***	0.287	***	0.448	***	0.325	***	0.263	0.019	0.174	0.141
**Dendritic cell**	HLA-DPB1	0.630	***	0.604	***	0.794	***	0.672	***	0.490	***	0.320	0.006
	HLA-DQB1	0.537	***	0.512	***	0.730	***	0.581	***	0.424	***	0.344	0.003
	HLA-DRA	0.646	***	0.621	***	0.819	***	0.711	***	0.461	***	0.276	0.018
	HLA-DPA1	0.651	***	0.631	***	0.786	***	0.674	***	0.475	***	0.315	0.007
	BCDA-1 (CD1C)	0.436	***	0.413	***	0.624	***	0.459	***	0.337	0.002	0.138	0.243
	BDCA-4 (NRP1)	0.436	***	0.465	***	0.413	***	0.340	***	0.076	0.503	0.112	0.346
	CD11c (ITGAX)	0.351	***	0.303	***	0.613	***	0.436	***	0.506	***	0.386	***
**Th1**	TBX21	0.578	***	0.593	***	0.877	***	0.806	***	0.427	***	0.278	0.017
	STAT4	0.188	***	0.159	***	0.789	***	0.689	***	0.369	0.001	0.198	0.093
	STAT1	0.531	***	0.529	***	0.682	***	0.611	***	0.066	0.563	0.119	0.315
	IFN-γ (IFNG)	0.350	***	0.332	***	0.792	***	0.692	***	0.395	***	0.285	0.015
	TNF-α (TNF)	0.171	***	0.135	0.004	0.656	***	0.489	***	0.397	***	0.232	0.048
**Th2**	GATA3	0.492	***	0.464	***	0.750	***	0.589	***	−0.225	0.046	−0.157	0.184
	STAT6	0.463	***	0.421	***	0.019	0.682	0.056	0.232	0.239	0.034	0.343	0.003
	STAT5A	0.426	***	0.368	***	0.243	***	0.296	***	0.399	***	0.346	0.003
	IL13	−0.083	0.068	−0.069	0.147	0.208	***	0.133	0.004	−0.158	0.164	0.021	0.862
**Tfh**	BCL6	−0.126	0.005	−0.104	0.028	0.372	***	0.319	***	0.073	0.521	0.200	0.089
	IL21	0.077	0.088	0.070	0.139	0.606	***	0.510	***	NA	NA	NA	NA
**Th17**	STAT3	0.422	***	0.425	***	0.342	***	0.353	***	0.217	0.055	0.285	0.015
	IL17A	0.034	0.453	0.025	0.596	−0.060	0.196	−0.136	0.004	0.213	0.059	0.119	0.317
**Treg**	FOXP3	0.035	0.449	0.050	0.293	0.763	***	0.631	***	0.190	0.094	0.183	0.120
	CCR8	0.245	***	0.250	***	0.792	***	0.705	***	0.392	***	0.302	0.009
	STAT5B	−0.086	0.057	−0.005	0.913	0.331	***	0.432	***	0.347	0.002	0.383	***
	TGFβ (TGFB1)	0.313	***	0.259	***	0.444	***	0.300	***	0.254	0.024	0.187	0.114
**Tex**	PD-1 (PDCD1)	0.594	***	0.577	***	0.833	***	0.737	***	0.420	***	0.203	0.086
	CTLA4	0.464	***	0.428	***	0.646	***	0.524	***	0.373	***	0.195	0.098
	LAG3	0.262	***	0.286	***	0.800	***	0.694	***	0.317	0.004	0.247	0.035
	TIM-3 (HAVCR2)	0.445	***	0.406	***	0.814	***	0.709	***	0.561	***	0.394	***
	GZMB	0.533	***	0.549	***	0.783	***	0.650	***	0.288	0.010	0.182	0.124

Numbers in red represent strong correlation: rho > 0.6; ***P < 0.001.

**Table 3:** Correlation analysis between CD96 and markers of CD8+ T cell, general T cell, DC, Th1, and Treg in GEPIA2.

Description	Markers	LGG				SKCM				ACC			
		Tumor		Normal		Tumor		Normal		Tumor		Normal	
		R	*P*	R	*P*	R	*P*	R	*P*	R	*P*	R	*P*
**CD8+ T cell**	CD8A	0.83	***	−0.01	0.900	0.87	***	0.66	***	0.49	***	0.61	***
	CD8B	0.85	***	0.18	0.063	0.87	***	0.53	***	0.44	***	0.53	***
**T cell**	CD3D	0.92	***	0.26	0.007	0.86	***	0.61	***	0.36	0.001	0.42	***
**(general)**	CD3E	0.96	***	0.55	***	0.86	***	0.68	***	0.43	***	0.58	***
	CD2	0.98	***	0.54	***	0.89	***	0.66	***	0.43	***	0.51	***
**Neutrophils**	CD66b (CEACAM8)	−0.02	0.700	0.27	0.005	−0.04	0.360	0.04	0.500	0.05	0.700	0.11	0.230
	CD11b (ITGAM)	0.51	***	0.19	0.047	0.46	***	0.28	***	0.41	***	0.11	0.230
	CCR7	0.88	***	0.54	***	0.42	***	0.14	0.015	0.59	***	−0.05	0.590
**DC**	HLA-DPB1	0.72	***	0.40	***	0.50	***	0.29	***	0.29	0.012	0.12	0.170
	HLA-DQB1	0.42	***	0.07	0.500	0.32	***	0.08	0.140	0.13	0.260	0.21	0.017
	HLA-DRA	0.72	***	0.35	***	0.54	***	0.27	***	0.34	0.002	0.08	0.400
	HLA-DPA1	0.69	***	0.42	***	0.41	***	0.26	***	0.24	0.033	0.14	0.110
**Th1**	TBX21	0.28	***	0.31	0.001	0.57	***	0.56	***	0.57	***	0.65	***
	STAT4	0.04	0.350	−0.10	0.330	0.63	***	0.46	***	0.57	***	0.28	0.002
	STAT1	0.31	***	−0.03	0.790	0.39	***	0.16	0.003	0.27	0.016	−0.11	0.200
	IFN-γ (IFNG)	0.67	***	0.04	0.660	0.54	***	0.49	***	0.39	0.001	0.24	0.006
	TNF-α (TNF)	0.09	0.034	−0.01	0.950	0.36	***	0.03	0.540	0.26	0.025	0.01	0.880
**Treg**	FOXP3	0.67	***	0.01	0.950	0.50	***	0.04	0.520	0.01	0.980	−0.07	0.440
	CCR8	0.80	***	0.10	0.290	0.48	***	0.32	***	0.01	0.910	0.01	0.880
	STAT5B	−0.01	0.850	0.33	0.001	0.22	***	0.20	***	0.27	0.019	−0.11	0.230
**Tex**	PD-1 (PDCD1)	0.69	***	0.40	***	0.55	***	0.39	***	0.32	0.004	0.50	***
	CTLA4	0.73	***	0.39	***	0.18	***	0.41	***	0.41	***	0.42	***
	LAG3	0.28	***	0.28	0.004	0.48	***	0.21	***	0.37	0.001	0.34	***
	TIM-3 (HAVCR2)	0.51	***	0.10	0.290	0.61	***	0.19	***	0.52	***	−0.04	0.650
	GZMB	0.51	***	0.04	0.660	0.44	***	0.57	***	0.51	***	−0.11	0.220

Numbers in red represent strong correlation: R > 0.6; ***P < 0.001.

## Discussion

In this report, we assessed the expression of CD96 in 33 different cancer types using the independent Oncomine and TIMER2.0 databases, revealing clear differences of pan-cancer CD96 expression between tumor and normal tissues. Oncomine data showed increased levels of CD96 in brain, breast, and kidney cancers and leukemia relative to normal tissues, while in several datasets, CD96 levels were lower in breast, colorectal and gastric cancers, as well as in leukemia, lymphoma, melanoma, and sarcoma. Analysis based on TCGA data showed that CD96 expression was increased in ESCA, HNSC, KIRC, KIRP, and STAD, but decreased in BRCA, COAD, LUSC, READ, SKCM, and THCA compared with adjacent normal controls. Mainly expressed on T cells and NK cells (29), CD96 expression level may reflect the abundance of these two immune infiltrates in TME indirectly. And we suppose that CD96 plays a delicate role in tumor initiation or development based on the differential expression profiles. However, considering the low protein expression of CD96, we do not recommend utilizing it as a molecular biomarker for tumor diagnosis.

Structurally, the CD96 molecule may play a contradictory role in immune processes. On the one hand, it has an immunoreceptor tyrosine-based inhibition motif (ITIM) motif, which is conserved in inhibit receptors such as KIR2DL (4); On the other hand, similar to the activating receptor NKG2D, CD96 harbors a YXXM motif (30). Therefore, whether CD96 activates NK cells to exert tumor cell killing effect or inhibits its activity is still inconclusive. The results of our analysis also corroborated this, as we consistently observed a correlation between elevated CD96 expression and a poorer GBM, LGG, and UVM prognosis. Meanwhile, CD96 demonstrated a protective effect in BLCA, HNSC, SKCM, and THYM. Especially, the impacts that CD96 exerts in LGG and SKCM were significant (*P* < 0.001). Together, these results indicated a malignant biological property and complicated prognostic value for CD96 in pan-cancer.

Another key finding of this study is that the CD96 expression is highly associated with immune infiltration. CD96 expression is positively correlated with the abundance of immune infiltrates, especially CD8+ T cells, DCs, macrophages, Tregs, and Tfh in various cancers. Existing evidence has suggested that TIGIT can inhibit the function of NK cells, thereby suppressing the deteriorating effects against tumor cells (31, 32). Besides, TIGIT^+^ Treg suppressed Th1/17 immunity, but not Th2 immunity (7, 33), in which interleukin 4 (IL-4) produced by Th2 cells promotes the differentiation of TAM toward M2 macrophage, leading to an immunosuppressive phenotype (34). Therefore, we supposed that CD96 could mediate similar immune functions in cancers. On the one hand, it may act as a costimulatory molecule, mediating the immunosuppression of NK and T cells. On the other hand, CD96 and its related molecules may participate in the process of immune adhesion and antigen presentation between DCs and T cells by affecting the binding of cytokines-cytokine receptors. Obviously, further work will be necessary in order to establish whether CD96 exerts such functions.

CD96 plays significant but contradictory roles in different cancers: it’s a distinctive risk and protective factor for LGG and SKCM, respectively. Subsequent analysis revealed increased expression of CD96 in higher grade glioma in TCGA-LGG cohort [consistent with our previous study (35)], but in lower stage melanoma in TCGA-SKCM cohort, showing significantly different expression distributions in LGG and SKCM. This suggests that CD96 can affect patient prognosis by influencing cancer malignant characteristics. And this effect is supposed to be achieved *via* influencing immune processes, since in LGG, CD96 related genes participate in the negative regulation of leukocyte. But in SKCM, CD96 related genes were mainly involved in the positive immune processes, such as lymphocyte activation, NK cell activation, and positive regulation of cytokine production. In the control cohort, CD96 expression was not associated with patient survival or any typical immune processes, either. These results strongly suggest that CD96 participates in the different immune processes and exerts different, even completely opposite effects on the tumor-related immunity and the patient prognosis. Notably, further analysis showed that CD96 in SKCM, but not in LGG, was positively associated with Th1 markers, again corroborating that CD96 participates in Th1 activation, thereby enhancing tumor inhibiting effects and prolonging patient survival time in SKCM, again suggesting CD96 impacted patient survival in an immunity-depended manner. Although the unique infiltration of immune cells in different tumors may affect our analysis results, we have reason to speculate that CD96 can influence the fate of immune infiltrates in TME, and may alter their distribution and subsequent interactions with malignancy cells, leading to distinct survival outcomes for different cancer patients.

There are some limitations to this study. Firstly, there is no experimental validation of the predicted results, and further studies should pay attention to the experimental validation of the predicted results by different methods, for example by reverse transcription-polymerase chain reaction (RT-PCR), immunohistochemistry, and immunocytochemistry. In addition, since high expression of CD96 is associated with diverse immune responses and controversial survival outcomes. It is necessary to further explore the hypothesis by examining CD96 protein levels using a large sample size to confirm the role of CD96 in different cancers. Moreover, we mainly employed TCGA database to perform these analyses, the included studies did not cover all previous published literatures involved CD96 and certain cancers, for instance, CD96 did not significantly related to LGG patient survival in GEO datasets by Prognoscan site. Therefore, experimental and clinical validation of the predicted results is still needed to confirm it clearly.

In summary, we applied integrated bioinformatics approaches to suggest that CD96 expression may mediate immune infiltration and impact patient prognosis in pan-cancer, sharing the potential as a prognostic biomarker and providing a novel direction to explore the pathogenesis malignance of these prevailing cancers. We concluded that CD96 was highly involved in the various immune responses and infiltration, shedding light to a new avenue where immunotherapies combining CD96 blockade and existing checkpoint inhibitors might be a feasible approach to suppressing these unpleasing tumors, especially gliomas in which CD96 is a distinctive risk factor.

## Methods

### Data Source and Processing

The Cancer Genome Atlas (TCGA; http://cancergenome.nih.gov) is a landmark cancer genomics program, which has molecularly characterized over 20,000 primary cancer and matched normal samples spanning 33 cancer types until Oct, 2020. Using UCSC Xena (https://xenabrowser.net/), we collected CD96 data from various cancer samples in the TCGA database (36). Fragments per kilobase million (FPKM) values were transformed into transcripts per kilobase million (TPM) values, which are more comparable between samples. Genotype-tissue expression (GTEx; http://commonfund.nih.gov/GTEx/) provides publicly available gene expression data from 54 normal tissue sites across nearly 1,000 people by RNA sequencing. Normal samples from both TCGA and GTEx (http://commonfund.nih.gov/GTEx/) databases were used for comparisons between cancer and normal tissue.

### CD96 Expression Profiles

HPA (https://www.proteinatlas.org/) is a program for mapping human proteins in cells, tissues and organs using integration of various omics technologies (37, 38). Therefore, we used HPA database to illustrate CD96 mRNA distribution among normal and cancer tissues. In addition, we obtained the immunohistochemistry images of CD96 proteins in the tissue atlas and pathology atlas panels.

Oncomine (www.oncomine.org) provides solutions for researchers with robust, peer-reviewed analysis methods and a powerful set of analysis functions that compute gene expression signatures, automatically extracting biological insights from the data based on published papers. We set threshold fold change as 1.5, and define P-value cutoff of 0.05 as significant to evaluate CD96 expression differences between cancers and adjacent normal tissues.

### Survival Analysis

The PrognoScan database (http://dna00.bio.kyutech.ac.jp/PrognoScan/index.html) is designed to facilitate meta-analyses of gene prognostic value by comparing the relationship between gene expression and relevant outcomes in a wide range of published cancer microarray data sets (39). We therefore utilized this database to assess the relationship between CD96 expression and patient outcomes in different cohorts. And its prognostic value was further analyzed in the TCGA dataset, as we performed survival analysis, computed the log-rank P value and hazard ratio (HR) with 95% confidence intervals (95% CI) using “survival” package in R. The results were displayed as forestplots (using “forestplot” package in R) and survival curves.

### Mutation Profiles

The cBioPortal for cancer genomics (http://www.cbioportal.org) is an open-access repository of cancer genomics datasets (40, 41). We investigated the copy number alteration (CNA) and mutation landscape of CD96 in pan-cancer. Catalog of Somatic Mutations in Cancer (COSMIC; https://cancer.sanger.ac.uk/cosmic/) is the largest and most comprehensive resource for exploring the impact of somatic mutations in human cancers (42). In this study, COSMIC was employed to investigate the specific mutation types of CD96 in various human cancers, and the results are depicted in pie charts.

### Correlation Analysis

The Cancer Regulome Explorer (http://explorer.cancerregulome.org/) enables users to search, filter, and visualize analytical results generated from TCGA data and explore associations among heterogeneous features. We used it to display the expression of CD96 and its correlation with other variables in cancers on the chromosomic level. Only associations with |pairwise correlation| ≥ 0.4 and -log10 (P value) ≥ 10 were shown in the circos plots. Pearson analysis was performed to assess the correlations between CD96 and immune checkpoints (including but not limited to PD-L1, TIM-3, and CTLA4), as well as mismatch repair (MMR) proteins. The results were displayed as heatmaps using “pheatmap” package in R.

### Immune Infiltration

Tumor purity was assessed in 33 human cancers using “estimate” package. Specifically, immune and stromal score represented the abundance of immune and stromal components, respectively. ESTIMATE score was the sum of previous scores, representing tumor purity indirectly. The correlations of CD96 expression with these scores in different cancers were depicted as scatter plots. Tumor IMmune Estimation Resource 2.0 (TIMER2.0; http://timer.cistrome.org/) web server is a comprehensive resource for systematical analysis of immune infiltrates across diverse cancer types (43, 44). At first, we used it to study the differential expression of CD96 between tumor and adjacent normal tissues across all TCGA cohorts. We then explored the association between CD96 expression and immune infiltration based on several immune deconvolution algorithms (**Supplementary Table 5**). We also employed TISIDB (http://cis.hku.hk/TISIDB/) to assess whether CD96 had a significant expression difference between responders and non-responders to immunotherapy (e.g., anti-PD-L1 and anti-PD-1) (45). At last, we assessed how CD96 correlated with the markers for immune cell subsets including CD8+ T cells, total T cells, B cells, monocytes, tumor-associated macrophages (TAMs), M1 and M2 macrophages, neutrophils, NK cells, DCs, Th1 cells, type 2 helper T cell (Th2), Tfh cells, type 17 helper T cell (Th17), Tregs, and exhausted T cells. Correlations with significance were re-analyzed using Gene Expression Profiling Interactive Analysis 2 (GEPIA2; gepia2.cancer-pku.cn) (46, 47).

### Enrichment Analysis

Metascape (http://metascape.org) integrates more than 40 gene function annotation databases and supplies various visualization methods, allowing readily gene function analysis (48). Herein, we employed this database to perform enrichment analysis on CD96 related genes obtained from Linkedomics (http://www.linkedomics.org/; |Pearson’s rho| > 0.4, P < 0.001) (49). The analysis included gene ontology (GO) and Kyoto Encyclopedia of Genes and Genomes (KEGG) enrichment analysis. We set min overlap as 3, min enrichment as 1.5, and *P* 0.05 as significant.

### Statistical Analysis

The distribution of CD96 in cancer was using HPA site, the expression of CD96 in cancer was using the Oncomine, TIMER2.0 and matched GTEx databases. The survival curves were generated by PrognoScan and “survival” package in R with data from the TCGA database. The survival results were displayed with HR, 95% CI, and log-rank *P* values. The mutation and CNV profiles were analyzed by cBioportal and COSMIC. The immune infiltration was analyzed using TIMER2.0 site and “estimate” package. Student’s t test and analysis of variance (ANOVA) test were used for comparisons between 2 groups, and for comparisons among >2 groups, respectively. Pearson’s correlation analyses were used to gauge the degree of correlation between certain variables, with the following R/rho values being used to judge the strength of correlation: 0–0.19, “very weak”; 0.20–0.39, “weak”; 0.40–0.59, “moderate”; 0.60–0.79, “strong”; 0.80–1.00, “very strong.” P < 0.05 was the significance threshold in most analysis.

## Data Availability Statement

The original contributions presented in the study are included in the article/**Supplementary Material**. Further inquiries can be directed to the corresponding authors.

## Author Contributions

WY and CL performed the data analysis and interpreted the data. WY prepared the draft. FL performed the visualization and revised the draft. ZL and FC designed the research and supervised all the work. All authors contributed to the article and approved the submitted version.

## Funding

This work was supported by the National Natural Science Foundation of China under Grant No. 82001223, and the Natural Science Foundation for Young Scientist of Hunan Province, China (Grant No. 2019JJ50952).

## Conflict of Interest

The authors declare that the research was conducted in the absence of any commercial or financial relationships that could be construed as a potential conflict of interest.

## References

[1] Tang J Pearce L O’Donnell-Tormey J Hubbard-Lucey VM. Trends in the global immuno-oncology landscape. Nat Rev Drug Discov (2018) 17(12):922. 10.1038/nrd.2018.202 30361553

[2] Xin Yu J Hubbard-Lucey VM Tang J. Immuno-oncology drug development goes global. Nat Rev Drug Discov (2019) 18(12):899–900. 10.1038/d41573-019-00167-9 31780841

[3] Xin Yu J Hodge JP Oliva C Neftelinov ST Hubbard-Lucey VM Tang J. Trends in clinical development for PD-1/PD-L1 inhibitors. Nat Rev Drug Discov (2020) 19(3):163–4. 10.1038/d41573-019-00182-w 32127660

[4] Dougall WC Kurtulus S Smyth MJ Anderson AC. TIGIT and CD96: new checkpoint receptor targets for cancer immunotherapy. Immunol Rev (2017) 276(1):112–20. 10.1111/imr.12518 28258695

[5] Yu X Harden K Gonzalez LC Francesco M Chiang E Irving B. The surface protein TIGIT suppresses T cell activation by promoting the generation of mature immunoregulatory dendritic cells. Nat Immunol (2009) 10(1):48–57. 10.1038/ni.1674 19011627

[6] Kurtulus S Sakuishi K Ngiow SF Joller N Tan DJ Teng MW. TIGIT predominantly regulates the immune response via regulatory T cells. J Clin Invest (2015) 125(11):4053–62. 10.1172/jci81187 PMC4639980 26413872

[7] Joller N Lozano E Burkett PR Patel B Xiao S Zhu C. Treg cells expressing the coinhibitory molecule TIGIT selectively inhibit proinflammatory Th1 and Th17 cell responses. Immunity (2014) 40(4):569–81. 10.1016/j.immuni.2014.02.012 PMC4070748 24745333

[8] Chan CJ Martinet L Gilfillan S Souza-Fonseca-Guimaraes F Chow MT Town L. The receptors CD96 and CD226 oppose each other in the regulation of natural killer cell functions. Nat Immunol (2014) 15(5):431–8. 10.1038/ni.2850 24658051

[9] Blake SJ Stannard K Liu J Allen S Yong MC Mittal D. Suppression of Metastases Using a New Lymphocyte Checkpoint Target for Cancer Immunotherapy. Cancer Discov (2016) 6(4):446–59. 10.1158/2159-8290.Cd-15-0944 26787820

[10] Sun H Huang Q Huang M Wen H Lin R Zheng M. Human CD96 Correlates to Natural Killer Cell Exhaustion and Predicts the Prognosis of Human Hepatocellular Carcinoma. Hepatology (Baltimore Md) (2019) 70(1):168–83. 10.1002/hep.30347 30411378

[11] Chiang EY de Almeida PE de Almeida Nagata DE Bowles KH Du X Chitre AS. CD96 functions as a co-stimulatory receptor to enhance CD8(+) T cell activation and effector responses. Eur J Immunol (2020) 50(6):891–902. 10.1002/eji.201948405 32043568

[12] Als AB Dyrskjøt L von der Maase H Koed K Mansilla F Toldbod HE. Emmprin and survivin predict response and survival following cisplatin-containing chemotherapy in patients with advanced bladder cancer. Clin Cancer Res (2007) 13(15 Pt 1):4407–14. 10.1158/1078-0432.Ccr-07-0109 17671123

[13] Kim WJ Kim EJ Kim SK Kim YJ Ha YS Jeong P. Predictive value of progression-related gene classifier in primary non-muscle invasive bladder cancer. Mol Cancer (2010) 9:3. 10.1186/1476-4598-9-3 20059769 PMC2821358

[14] Lee JS Leem SH Lee SY Kim SC Park ES Kim SB. Expression signature of E2F1 and its associated genes predict superficial to invasive progression of bladder tumors. J Clin Oncol (2010) 28(16):2660–7. 10.1200/jco.2009.25.0977 20421545

[15] Li Y Zou L Li Q Haibe-Kains B Tian R Li Y. Amplification of LAPTM4B and YWHAZ contributes to chemotherapy resistance and recurrence of breast cancer. Nat Med (2010) 16(2):214–8. 10.1038/nm.2090 PMC2826790 20098429

[16] Wang Y Klijn JG Zhang Y Sieuwerts AM Look MP Yang F. Gene-expression profiles to predict distant metastasis of lymph-node-negative primary breast cancer. Lancet (London England) (2005) 365(9460):671–9. 10.1016/s0140-6736(05)17947-1 15721472

[17] Smith JJ Deane NG Wu F Merchant NB Zhang B Jiang A. Experimentally derived metastasis gene expression profile predicts recurrence and death in patients with colon cancer. Gastroenterology (2010) 138(3):958–68. 10.1053/j.gastro.2009.11.005 PMC3388775 19914252

[18] Freeman TJ Smith JJ Chen X Washington MK Roland JT Means AL. Smad4-mediated signaling inhibits intestinal neoplasia by inhibiting expression of β-catenin. Gastroenterology (2012) 142(3):562–71.e2. 10.1053/j.gastro.2011.11.026 22115830 PMC3343368

[19] Williams CS Bernard JK Demory Beckler M Almohazey D Washington MK Smith JJ. ERBB4 is over-expressed in human colon cancer and enhances cellular transformation. Carcinogenesis (2015) 36(7):710–8. 10.1093/carcin/bgv049 PMC4572918 25916654

[20] Chen MS Lo YH Chen X Williams CS Donnelly JM Criss ZK 2nd. Growth Factor-Independent 1 Is a Tumor Suppressor Gene in Colorectal Cancer. Mol Cancer Res MCR (2019) 17(3):697–708. 10.1158/1541-7786.Mcr-18-0666 30606770 PMC7387124

[21] Lee ES Son DS Kim SH Lee J Jo J Han J. Prediction of recurrence-free survival in postoperative non-small cell lung cancer patients by using an integrated model of clinical information and gene expression. Clin Cancer Res (2008) 14(22):7397–404. 10.1158/1078-0432.Ccr-07-4937 19010856

[22] Yoshihara K Tajima A Yahata T Kodama S Fujiwara H Suzuki M. Gene expression profile for predicting survival in advanced-stage serous ovarian cancer across two independent datasets. PLoS One (2010) 5(3):e9615. 10.1371/journal.pone.0009615 20300634 PMC2837379

[23] Bogunovic D O’Neill DW Belitskaya-Levy I Vacic V Yu YL Adams S. Immune profile and mitotic index of metastatic melanoma lesions enhance clinical staging in predicting patient survival. Proc Natl Acad Sci USA (2009) 106(48):20429–34. 10.1073/pnas.0905139106 PMC2787158 19915147

[24] Baretti M Le DT. DNA mismatch repair in cancer. Pharmacol Ther (2018) 189:45–62. 10.1016/j.pharmthera.2018.04.004 29669262

[25] Zhao P Li L Jiang X Li Q. Mismatch repair deficiency/microsatellite instability-high as a predictor for anti-PD-1/PD-L1 immunotherapy efficacy. J Hematol Oncol (2019) 12(1):54. 10.1186/s13045-019-0738-1 31151482 PMC6544911

[26] Yarchoan M Hopkins A Jaffee EM. Tumor Mutational Burden and Response Rate to PD-1 Inhibition. N Engl J Med (2017) 377(25):2500–1. 10.1056/NEJMc1713444 PMC6549688 29262275

[27] Sha D Jin Z Budczies J Kluck K Stenzinger A Sinicrope FA. Tumor Mutational Burden as a Predictive Biomarker in Solid Tumors. Cancer Discov (2020) 10(12):1808–25. 10.1158/2159-8290.Cd-20-0522 PMC7710563 33139244

[28] Samstein RM Lee CH Shoushtari AN Hellmann MD Shen R Janjigian YY. Tumor mutational load predicts survival after immunotherapy across multiple cancer types. Nat Genet (2019) 51(2):202–6. 10.1038/s41588-018-0312-8 PMC6365097 30643254

[29] Fuchs A Cella M Giurisato E Shaw AS Colonna M. Cutting edge: CD96 (tactile) promotes NK cell-target cell adhesion by interacting with the poliovirus receptor (CD155). J Immunol (Baltimore Md 1950) (2004) 172(7):3994–8. 10.4049/jimmunol.172.7.3994 15034010

[30] Georgiev H Ravens I Papadogianni G Bernhardt G. Coming of Age: CD96 Emerges as Modulator of Immune Responses. Front Immunol (2018) 9:1072. 10.3389/fimmu.2018.01072 29868026 PMC5966540

[31] Stanietsky N Simic H Arapovic J Toporik A Levy O Novik A. The interaction of TIGIT with PVR and PVRL2 inhibits human NK cell cytotoxicity. Proc Natl Acad Sci USA (2009) 106(42):17858–63. 10.1073/pnas.0903474106 PMC2764881 19815499

[32] Wang F Hou H Wu S Tang Q Liu W Huang M. TIGIT expression levels on human NK cells correlate with functional heterogeneity among healthy individuals. Eur J Immunol (2015) 45(10):2886–97. 10.1002/eji.201545480 26171588

[33] Kourepini E Paschalidis N Simoes DC Aggelakopoulou M Grogan JL Panoutsakopoulou V. TIGIT Enhances Antigen-Specific Th2 Recall Responses and Allergic Disease. J Immunol (Baltimore Md 1950) (2016) 196(9):3570–80. 10.4049/jimmunol.1501591 27016609

[34] Orecchioni M Ghosheh Y Pramod AB Ley K. Macrophage Polarization: Different Gene Signatures in M1(LPS+) vs. Classically and M2(LPS-) vs. Alternatively Activated Macrophages. Front Immunol (2019) 10:1084. 10.3389/fimmu.2019.01084 31178859 PMC6543837

[35] Liu F Huang J He F Ma X Fan F Meng M. CD96, a new immune checkpoint, correlates with immune profile and clinical outcome of glioma. Sci Rep (2020) 10(1):10768. 10.1038/s41598-020-66806-z 32612110 PMC7330044

[36] Goldman MJ Craft B Hastie M Repečka K McDade F Kamath A. Visualizing and interpreting cancer genomics data via the Xena platform. Nat Biotechnol (2020) 38(6):675–8. 10.1038/s41587-020-0546-8 PMC7386072 32444850

[37] Uhlén M Fagerberg L Hallström BM Lindskog C Oksvold P Mardinoglu A. Proteomics. Tissue-based map of the human proteome. Science (New York NY) (2015) 347(6220):1260419. 10.1126/science.1260419 25613900

[38] Uhlen M Zhang C Lee S Sjöstedt E Fagerberg L Bidkhori G. A pathology atlas of the human cancer transcriptome. Sci (New York NY) (2017) 357(6352):eaan2507. 10.1126/science.aan2507 28818916

[39] Mizuno H Kitada K Nakai K Sarai A. PrognoScan: a new database for meta-analysis of the prognostic value of genes. BMC Med Genomics (2009) 2:18. 10.1186/1755-8794-2-18 19393097 PMC2689870

[40] Cerami E Gao J Dogrusoz U Gross BE Sumer SO Aksoy BA. The cBio cancer genomics portal: an open platform for exploring multidimensional cancer genomics data. Cancer Discov (2012) 2(5):401–4. 10.1158/2159-8290.Cd-12-0095 PMC3956037 22588877

[41] Gao J Aksoy BA Dogrusoz U Dresdner G Gross B Sumer SO. Integrative analysis of complex cancer genomics and clinical profiles using the cBioPortal. Sci Signaling (2013) 6(269):pl1. 10.1126/scisignal.2004088 PMC4160307 23550210

[42] Tate JG Bamford S Jubb HC Sondka Z Beare DM Bindal N. COSMIC: the Catalogue Of Somatic Mutations In Cancer. Nucleic Acids Res (2019) 47(D1):D941–d7. 10.1093/nar/gky1015 PMC6323903 30371878

[43] Li T Fan J Wang B Traugh N Chen Q Liu JS. TIMER: A Web Server for Comprehensive Analysis of Tumor-Infiltrating Immune Cells. Cancer Res (2017) 77(21):e108–e10. 10.1158/0008-5472.Can-17-0307 PMC6042652 29092952

[44] Li T Fu J Zeng Z Cohen D Li J Chen Q. TIMER2.0 for analysis of tumor-infiltrating immune cells. Nucleic Acids Res (2020) 48(W1):W509–w14. 10.1093/nar/gkaa407 PMC7319575 32442275

[45] Ru B Wong CN Tong Y Zhong JY Zhong SSW Wu WC. TISIDB: an integrated repository portal for tumor-immune system interactions. Bioinf (Oxford Engl) (2019) 35(20):4200–2. 10.1093/bioinformatics/btz210 30903160

[46] Tang Z Li C Kang B Gao G Li C Zhang Z. GEPIA: a web server for cancer and normal gene expression profiling and interactive analyses. Nucleic Acids Res (2017) 45(W1):W98–w102. 10.1093/nar/gkx247 28407145 PMC5570223

[47] Tang Z Kang B Li C Chen T Zhang Z. GEPIA2: an enhanced web server for large-scale expression profiling and interactive analysis. Nucleic Acids Res (2019) 47(W1):W556–w60. 10.1093/nar/gkz430 PMC6602440 31114875

[48] Zhou Y Zhou B Pache L Chang M Khodabakhshi AH Tanaseichuk O. Metascape provides a biologist-oriented resource for the analysis of systems-level datasets. Nat Commun (2019) 10(1):1523. 10.1038/s41467-019-09234-6 30944313 PMC6447622

[49] Vasaikar SV Straub P Wang J Zhang B. LinkedOmics: analyzing multi-omics data within and across 32 cancer types. Nucleic Acids Res (2018) 46(D1):D956–d63. 10.1093/nar/gkx1090 PMC5753188 29136207

