# Supplementary material for: CD96 Correlates With Immune Infiltration and Impacts Patient Prognosis: A Pan-Cancer Analysis
Source: Front Oncol. 2021 Feb 19;11:634617. doi: 10.3389/fonc.2021.634617 (PMC7935557; doi:10.3389/fonc.2021.634617)
Supplement: Supplementary file 1 [file DataSheet_1.docx]

Supplementary Material


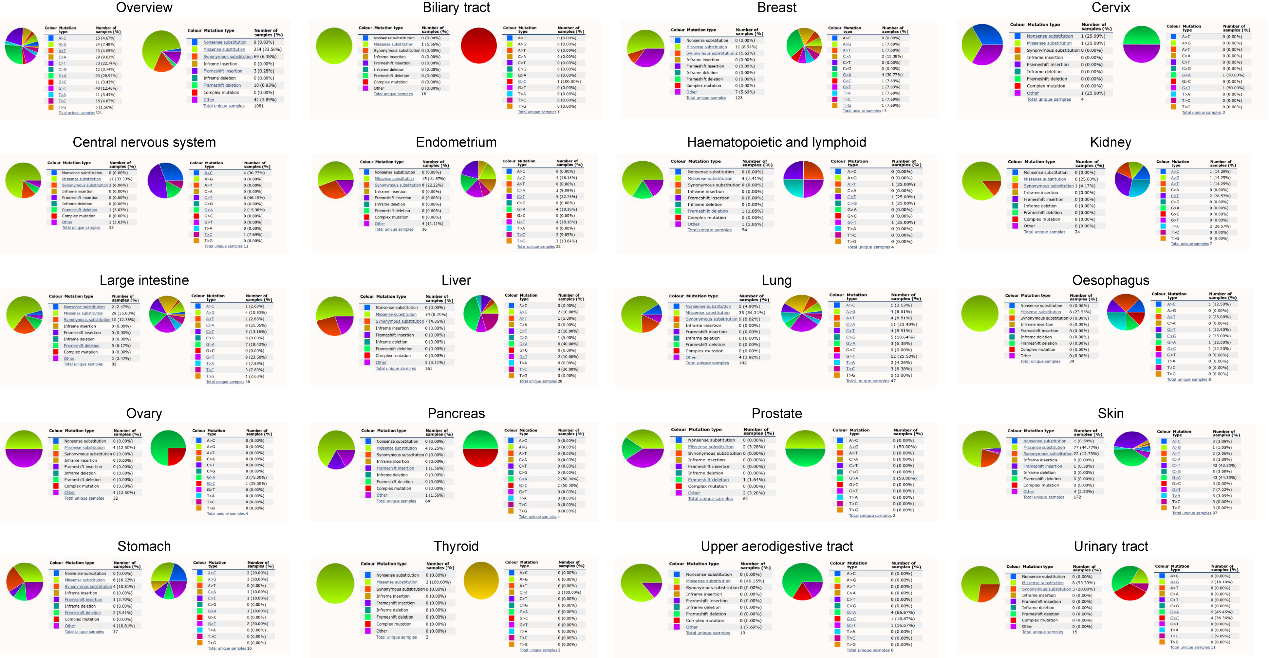


**Supplementary Figure 1.** Pie chart showing the percentage of the different mutation types of CD96 in human cancers according to the COSMIC database.
